# A Clinical Feasibility Study of the Forensic Psychiatry and Violence Oxford (FoVOx) Tool

**DOI:** 10.3389/fpsyt.2019.00901

**Published:** 2019-12-13

**Authors:** Robert Cornish, Alexandra Lewis, Owen Curwell Parry, Oana Ciobanasu, Susan Mallett, Seena Fazel

**Affiliations:** ^1^Department of Psychiatry, University of Oxford, Oxford, United Kingdom; ^2^Thames Valley Forensic Mental Health Service, Oxford Health NHS Foundation Trust, Oxford, United Kingdom; ^3^Broadmoor Hospital, West London NHS Trust, Southall, United Kingdom; ^4^Institute of Applied Health Research, University of Birmingham, Birmingham, United Kingdom

**Keywords:** FoVOx, risk assessment, feasibility, recidivism, secure hospital, forensic psychiatry

## Abstract

**Background:** Risk assessment informs decisions around admission to and discharge from secure psychiatric hospital and contributes to treatment and supervision. There are advantages to using brief, scalable, free online tools with similar accuracy to instruments currently used. We undertook a study of one such risk assessment, the Forensic Psychiatry and Violence Oxford (FoVOx) tool, examining its acceptability, feasibility, and practicality.

**Methods:** We completed the FoVOx tool on all discharges from six secure psychiatric hospitals in one region in England over two years. We interviewed 11 senior forensic psychiatrists regarding each discharge using a standardized questionnaire. Their patient’s FoVOx score was compared to clinical risk assessment, and the senior clinicians were asked if they considered FoVOx scores accurate and useful. A modified thematic analysis was conducted, and clinicians were surveyed about current risk assessment practice on discharge.

**Results:** Of 90 consecutive discharges, 84 were included in the final analysis. The median FoVOx probability score was 11% risk of violent recidivism in two years after discharge. We estimated that 12 (14%) individuals reoffended since discharge; all were in the medium or high risk FoVOx categories. Clinical assessment of risk agreed with the FoVOx categories in around half the cases. Clinicians were more likely to provide lower risk categories compared with FoVOx ones. FoVOx was considered to be an accurate representation of risk in 67% of cases; clinicians revised their view on some patient’s risk assessment after being informed of their FoVOx scores. Completing FoVOx was reported to be helpful in the majority of cases. Reasons included improved communication with other agencies, reassurance to clinical teams, and identifying additional factors not fully considered. 10 of the 11 respondents reported that FoVOx was practical, and seven of 11 reported that they would use it in the future, highlighting its brevity and speed of use compared to existing risk assessment tools.

**Conclusions:** Senior clinicians in this regional forensic psychiatric service found the FoVOx risk assessment tool feasible, practical, and easy to use. Its use addressed a lack of consistency around risk assessment at the point of discharge and, if used routinely, could assist in clinical decision-making.

## Introduction

After discharge from forensic psychiatric hospital, rates of violent reoffending are reported to range from 2% to 8% per year in high income countries, and one cohort study based on around half of the forensic hospitals in England reported that 1 in 8 men and 1 in 16 women were convicted of serious offences over a mean follow-up of 6 years (1). Thus, risk assessment has become an integral part of forensic mental health in order to inform decisions about admission, management, and discharge (2). Further, professionals working in forensic mental health regularly advise court proceedings, which can involve considerations of future risk.

If accurately done, risk assessment should identify those patients presenting with the highest risk, reduce length of stay, and assist in treatment allocation. Structured violence risk assessment is broadly split into two approaches: actuarial tools, which use statistical methods to give a population-based percentage chance of reoffending, and structured professional judgment tools, which attempt to guide mental health professionals by identifying some risk factors. Structured professional judgment tools are more frequently used in clinical settings. In the UK, for example, 90% of medium secure units report using them in one survey (3), and their completion is used as a key performance indicator (4). However, there are important problems with using them (5). Structured professional judgment tools take a long time to complete, for example 15 person-hours to complete an initial HCR-20 (6). They often have low to moderate validity in field studies (7), have often been developed in prison, rather than hospital, settings, and using methods to derive them which are dated. Further, there have been low standards in reporting, including few performance measures, authorship bias (8), wide variations in what constitutes ‘high’ risk (9), and their underlying risk factors are based on heterogeneous samples and do not incorporate new evidence on risk (10). In the case of the commonly-used HCR-20 and PCL-R, for example, it has been found that most of the factors are not predictive (11). One new approach has been to use solely dynamic risk factors, but this may lead to harsher penalties for minority groups by conflating risk with rehabilitative needs (12) and also poor accuracy as strong risk factors for reoffending including sex, age, and criminal history are omitted. Thus, the potential use of high-quality actuarial tools needs reconsideration in forensic mental health (13).

One such tool is the Forensic Violence Oxford (FoVOx) tool (14), which was developed using all forensic psychiatric patients in Sweden and based on the largest forensic psychiatric sample to date. When reported, the FoVOx study was novel in that it incorporated independent risk factors tested in a large sample, reported calibration (observed vs. expected probabilities) and published a study protocol. The FoVOx tool also has the advantage of using routinely available data, which are less liable to bias than interview-based measures (e.g. of a personality trait). The 12 items within the FoVOx tool are sex, age, previous violent crime, previous serious violent crime, primary discharge diagnosis, drug use disorder at point of hospitalization or discharge, any lifetime drug use disorder, alcohol use disorder at point of hospitalization or discharge, personality disorder at discharge, employment at admission, five or more prior inpatient episodes, and whether current length of stay has exceeded one year. The FoVOx tool is scalable, quick, free to use and available online. All the model coefficients are reported, meeting a key concern of using clinical prediction models that they should be transparent. In the derivation sample, the AUC was 0.77, which makes it as accurate in terms of discrimination as existing tools (15). Its use could enable clinicians to provide a reasonably accurate risk assessment in a brief and cost-effective way, and free up time to focus on clinical care and risk management rather than risk assessment. Possible limitations of the FoVOx tool are that it does not specifically predict serious (as opposed to any) violent reoffending and has not been externally validated to date.

In addition to external validation, prior to introducing any new risk assessment into a clinical setting, information about potential users and their decision-making is necessary (16). Clinical impact should be assessed, including where it could sit in the clinical pathway and the consequences of its use. Attitudes towards any tool should be sought, and any preconceptions about risk prediction models identified. Therefore, we undertook a feasibility study of the FoVOx tool, assessing its acceptability to professionals, demand for its use, and its practicality in one regional English forensic psychiatric service.

## Methods

The study protocol used a mixed methods approach by identifying discharged patients and scoring them using the FoVOx tool at the point of their discharge, which was followed up by qualitative work assessing clinician views about the use of the FoVOx tool.

### Sample

#### Patients

We identified all consecutive patients discharged from the Thames Valley Forensic Mental Health Services between March 2016 and March 2018, covering three counties (Oxfordshire, Buckinghamshire and Berkshire) across different levels of security (medium and low security, and a pre-discharge unit). All patients, both male and female, were included in the study irrespective of diagnosis or any other individual factor. All were over the age of 18. If any patients were discharged more than once from the service during that time, the most recent discharge was selected.

#### Clinicians

We interviewed all the senior clinicians (‘Responsible Clinicians’) in the service, made up of eight men and three women. In England and Wales, Responsible Clinicians are the legally considered the lead professionals involved in the care of detained forensic mental health patients. All Responsible Clinicians in the service were consultant (i.e. certified on the General Medical Council Specialist Register) forensic psychiatrists.

### Measures

#### FoVOx

Two members of the study team (AL and OP) accessed the electronic healthcare record (‘Care Notes’) of the discharged patients to obtain the information required to calculate their FoVOx score at the point of the most recent discharge, using an online calculator available at https://oxrisk.com/fovox/. 

#### Questionnaire

A standardized tool was developed to collect views and information from senior clinicians (**Appendix 1**). Each clinician underwent an in-depth interview by one of the study team (RC, OP, or AL) regarding each discharge. The standardized tool contained no patient identifiable information. The anonymized discharge number was the only identifier. During the interviews, patients’ identities and discharge location were shared in order to collect the clinician view on risk assessment of their own patients.

The clinician was asked to provide their estimate of the 2-year risk of a violent conviction (meaning any interpersonal violent or sexual offence) at the point of their most recent discharge. They were asked to provide a high, medium or low risk rating in line with pre-specified FoVOx categories): Low (< 5% chance of violent offending within two years of discharge), Medium (5–20%), High (> 20%) or state if they could not recall this. They were then asked if, according to their knowledge, their patient had committed a violent offence since that period of discharge.

After this, the patient’s FoVOx score (both probability and categorical) at the time of discharge was shared with the Responsible Clinician. Cohen’s Kappa was calculated for agreement between clinical categorical risk assessment and FoVOx category. The clinician was then asked if they considered this to be a fair representation of their risk and if not, why. Participants were also asked whether it would have been of benefit to know the FoVOx probability and categorical score at the point of discharge, for example by altering clinical management at that point. Reasons were again given for each case. Answers to the two questions about whether FoVOx was accurate and useful were recorded, using the interviewee’s wording and with an opportunity for the clinician interviewed to confirm that the transcribed notes represented their stated reasons. One researcher (RC) analyzed these records, creating individual response codes, noting how often these were each stated and thematically grouped them. The transcripts were then read by a second researcher (OC), who re-analyzed according to themes independently, before the two researchers met to agree a consensus about the principal themes.

The clinician was next asked whether they routinely use a risk assessment tool at discharge in order to check whether they were already using the FoVOx tool and to determine whether they were using other tools they consider effective.

The unpopulated FoVOx tool was then shown to the clinician. They were asked their views on its practicality, ease of use, and future plans for risk assessment. Participants were asked to give specific reasons as to whether they thought FoVOx was practical to use, and whether they would use FoVOx in the future. Again, these responses were recorded and coded and grouped into themes by two researchers (RC and OC).

### Ethics

The project was approved by the Oxford Health NHS Foundation Trust Clinical Governance Committee in March 2018 and by the Clinical Lead for Forensic Services as a Service Evaluation project. Therefore, individual informed consent was not deemed necessary. No data beyond that collated in routine clinical care was used, and the management of patients was not impacted by the study. To identify patients, existing discharge data being collected by the Trust for audit purposes was used. All Responsible Clinicians participated in the study voluntarily, and patient data was anonymized other than for the ‘unblinding’ during the Responsible Clinician interviews.

## Results

### Sample

**Figure 1:**
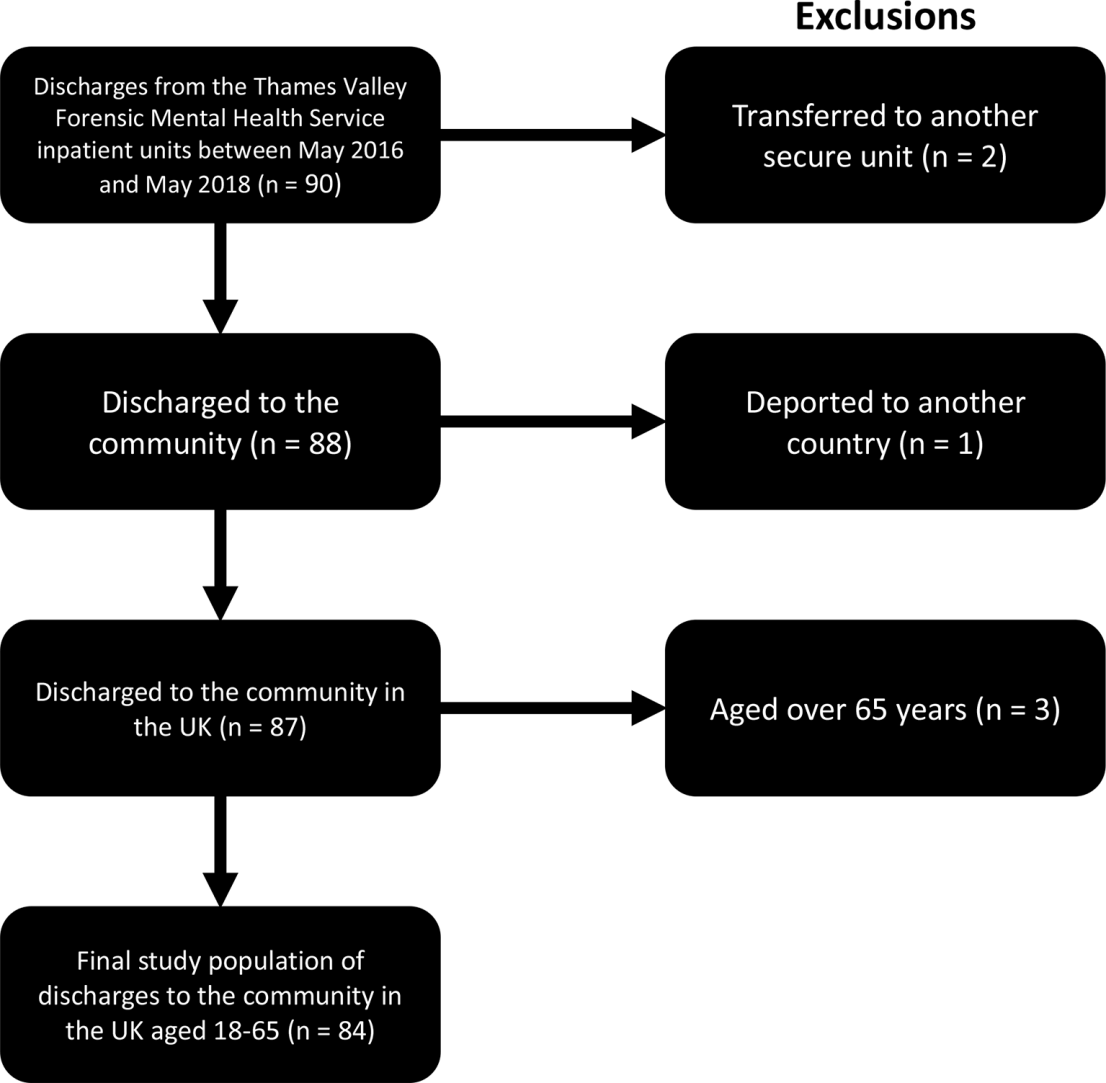
Inclusion of discharged patients from secure hospital into study.

Ninety discharges from forensic psychiatric hospitals were identified from May 2016 to May 2018. Six patients were excluded from analysis (two had been transferred to another secure psychiatric setting, one deported abroad and three were aged over 65 years). Thus, 84 patients were included in the study (**Figure 1**). Of these, 11 were female (13%). One transgender patient had their assigned gender used for FoVOx scoring (17). The mean age of patients was 39.2 years, SD 10.7, range 21–60.

The number of discharged patients per clinician was 2 to 18. The median number of days from discharge to study interview was 485 (interquartile range 339–643). Sample characteristics and FoVOx scores are included in **Table 1**. 9 of the 11 clinicians (82%) reported that they routinely completed a risk assessment process around the time of discharge. These included a multi-disciplinary clinical risk assessment, a description of risk in a Mental Health Review Tribunal Report, the patient’s latest HCR-20, and HoNOS (Health of the Nation Outcome Scales). One respondent reported regularly using a structured risk assessment specifically at the point of discharge.

**Table 1:** Sample characteristics and FoVOx scores.

Demographics		
	Male:female Age at discharge (SD)	73:11 39.2 (10.7)
	Median days since discharge (IQR)	485 (334–643)
	Median FoVOx score (range)	11% (2%–49%)
**FoVOx categories**	All included patients	Violent recidivists (based on clinician recall)
-Low	12 (14%)	0
-Medium	55 (66%)	8 (67%)
-High	17 (20%)	4 (33%)
**Responsible clinician view on FoVOx**	Yes	No
-Accurate?	56 (67%)	28 (33%)
-Helpful?	49 (58%)	35 (42%)

### FoVOx Scores

FoVOx scores were calculated from clinical records and took no more than 15 minutes per case. The median FoVOx probability score was a 11% chance of violent recidivism in 2 years (interquartile range 6–19%, range 2–49%). In terms of categories, 12 (14%) were low risk, 55 (66%) medium risk and 17 (20%) high risk. Some individual items were missing for 15 patients, and for these a FoVOx risk score range was generated (as per the online calculator). Of these, four ranges crossed low-medium or medium-high categories. In these cases, the higher risk rating was presented to the clinician.

### Recidivism

Of the 84 patients included, five were lost to follow up. Of the remaining 79 who were still in contact with mental health services, 12 (14%) were reported to have committed a violent offence since discharge based on information known to the Responsible Clinicians. Of these, eight had FoVOx scores in the medium category and four were in the high category.

### Concordance Between FoVOx Scores and Clinical Judgment (Case by Case)

There was agreement for risk assessment based on three categories (i.e. low/medium/high) between the clinical unstructured judgment and the FoVOx score in 49% of cases (41 out of 84; κ = 0.20 [95% CI, 0.06–0.35]). Where there was disagreement, clinicians were more likely to score patients in a lower risk category compared with their FoVOx score (in 36 of the 43 cases). When considering two risk categories: low and medium/high, there was agreement between clinical unstructured judgment and the FoVOx score in 63% of cases (53 out of 84; κ = 0.22 [95% CI, 0.06–0.38]). For most cases (56 out of 84, 67%), the FoVOx score (combined categorical and probability) was felt by the clinician to be an accurate representation of the violence risk at discharge. In the 28 cases (33%) where it was not, the clinician was asked to give reasons. Two main themes were identified. The first was that clinicians viewed certain risk factors as dynamic and modifiable by treatment, such as substance abuse and the response to medication and psychological treatment; “*The dynamic risk factors have been modified in hospital, there was no alcohol or drug use on discharge and these were relevant for previous offending*” and “*The risk is too high, it doesn’t take into account any completed therapeutic intervention.*” These were thought to contribute to risk in both directions; if the patient had responded to treatment the FoVOx score was felt to be an overestimate, and vice versa. The second theme was that clinicians felt that there were additional factors which influenced risk which were not measured by the FoVOx tool. These included the recency of offending and violence, and the nature of supervision in the community, as well as cases in which the patient was only felt to pose a risk in a specific set of circumstances: *“Risk is over-represented as there has not been any violence for the past 15 years*”, *“The estimated risk is too high; the patient has engaged very well with treatment and supervision in the community*” and *“This tool underestimates risk as relationship instability is a risk factor”*. These themes are summarized in **Table 2**.

**Table 2:** Qualitative feedback on challenges with FoVOx scoring.

Theme	Sub theme	FoVOx score is too high	FoVOx score is too low
**Dynamic Risk Factors**	Primary discharge diagnosis—medical treatment	Good response to medication	Poor response to medication
	Personality disorder diagnosis—psychological treatment	Successful (increased insight, specific work on offence)	Unsuccessful (non-engagement, untreated personality disorder)
	Substance misuse diagnosis	No longer using substances	High risk of substance misuse after discharge
**Risk factors not identified by FoVOx**	Supervision	Engaged with community mental health support, use of statutory supervision	Uncooperative with supervision
	Chronicity of violence	No violence in hospital	Frequent violence in hospital
	Psychosocial support	Improved relationships with family, good psychosocial functioning, lifestyle change	Relationship instability
	Specific circumstances to index offence	No forensic history prior to index offence, long period of time since index offence, offending could only occur in a specific context	Lengthy past forensic history, unpredictability

### Views on Utility At the Point of Discharge (Case by Case)

Responsible Clinicians reported that it would have been helpful to know FoVOx scores at the point of discharge in 49 (58%) cases, and not that helpful in 35 (42%) cases. Qualitative feedback is summarized in **Table 3**. The most frequent reasons given for why FoVOx would be helpful were related to its concordance with existing clinical risk assessment, including supporting and providing further evidence of the clinical categorization of risk if it aligned with the FoVOx score; *“(It) can offer confirmation of informal risk assessment”*. Improved information sharing with other agencies was repeatedly noted, including being used as an additional source of evidence; “*this was a high-profile case and the Tribunal was reluctant to discharge, perhaps the clinical view over-estimated risk and it would have been helpful to have the FoVOx score*”. It was also reported, for 14 cases, that using FoVOx would identify risk factors which had not been fully considered by clinicians, suggesting that it could impact on risk management in addition to risk assessment; “*Can confirm risk assessment and help identify and consider other risk factors*” and “*Identifies outstanding areas of risk*”. In cases where FoVOx was considered not useful, the most common reason given was clinicians not attributing value to any actuarial tool; “*Won’t add value to clinical practice and is unlikely to add to discharge planning. Doesn’t use any clinical risk factors, such as insight*”. Other comments included that it might lead to unintended consequences. For example, FoVOx could increase anxiety if it rated patients at higher risk than clinical assessment. It was also reported that sharing the FoVOx score could lead to delays in liaison with other agencies, for example if they refused or delayed housing or support on discharge on the basis of high risk; *“Can increase anxiety and delay discharge planning, induce self-doubt in clinical decision-making, make other teams reluctant to take over care.”*

**Table 3:** Qualitative feedback on the usefulness of FoVOx scoring.

Theme	Sub Theme	Helpful	Unhelpful
**Used as evidence to support decision-making**	As part of discharge planning	Other agencies more likely to support discharge, e.g. Mental Health Review Tribunals, Parole Board	Could lead the same agencies less likely to discharge, FoVOx score is less relevant if patient is discharged due to circumstances other than a reduction in risk
	In liaison with third parties	Improved information sharing with accommodation providers, non-forensic mental health services, probation and MAPPA	Negative responses such as not accepting patient for housing.
**Risk assessment**	Reassurance	Reassurance if agrees with clinical assessment, reducing anxiety if FoVOx rates risk lower than clinician	No added value if FoVOx and clinician assessment agree, increasing anxiety and leading to review if FoVOx rates risk higher than clinician
	Changing patient management	Identification of unaddressed risk factors and informing management decisions such as threshold to recall	Over or under-estimates risk due to reliance on historical factors
	Existing perceptions of risk assessment	Highlights over-reliance on clinical factors as being predictive of recidivism	Skepticism about the value of any actuarial tool
	Need to differentiate between serious and less serious offending		Inability of FoVOx to predict serious, as opposed to any, violent recidivism
	Discharge due to factors other than risk reduction		Discharge was dictated by factors other than a reduction in risk.

### Overall Opinion of Practicality and Future Use

After reviewing individual discharges, 10 (90%) clinicians reported that the FoVOx tool is practical to use and that it could be completed without reference to medical notes. All respondents could complete FoVOx scoring in under 1 minute for their most recent discharged patient.

Seven of 11 (64%) respondents reported that they would use FoVOx in the future for a number of reasons (**Table 4**). In addition to improved information sharing and possible impact on management, there were a range of positive comments about the FoVOx tool specifically. These included the ease with which information could be found, and the speed with which it could be completed. Criticisms included the emphasis on static, historical factors, inability to specifically predict serious (as opposed to any) violence, and a preference to see validation studies in a UK forensic sample before local adoption.

**Table 4:** Qualitative feedback on whether clinicians would use FoVOx in the future.

	Reasons for using	Reasons for not using
**FoVOx specific**	Information is easy to find	Based on static, historical risk factors
		No actuarial tool is of value
	Information can be found quickly	May narrow thinking about risk assessment
	Useful adjunct to existing risk assessment	Not wishing to add another tool to existing metrics
		Lack of sensitive clinical risk factors (e.g. insight, response to medication)
	Has construct validity	Not yet validated in a UK forensic population
		Inability to predict serious, as opposed to any violence
**Information sharing**	Information sharing with other agencies	
	Resolves disagreements about risk	
**Impact on management**	Reassurance when agrees with clinical opinion	No added value if agrees with existing risk assessment
		May provide false reassurance
	Helpful challenge when disagrees with clinical opinion	
	Guides community management (e.g. level of supervision)	

Finally, clinicians were asked if they had any other comments regarding the FoVOx tool. Its potential use at an earlier stage, either at gatekeeping or to screen referrals, was noted twice. The benefits of its brevity and speed of use over existing actuarial tools were repeatedly noted, even among clinicians who felt that actuarial risk assessment tools were of little value: *“If we have to use an actuarial tool then I would use this*” and “*Straightforward, reassuring, also helpful/interesting to consider cases where there is discrepancy. Nothing to lose, why wouldn’t you?*”. Three respondents stated that the low/medium/high risk categories were unnecessary. There were also some specific queries about individual cases, including whether those in full-time education at the time of their index offence should be considered as employed, and classifying some UK offences as serious or aggravated/otherwise.

## Discussion

We examined the use of a novel violence risk assessment tool (FoVOx) on 84 consecutive discharges from secure (forensic) psychiatric hospitals within one region of England over 2 years to assess its feasibility and acceptability. As part of this, individual interviews were conducted with senior clinicians regarding each discharge to assess the potential impact of the FoVOx tool on risk assessment and management.

We found that the data required to complete the FoVOx tool was routinely collected, and there was no need to seek additional sources of information other than the patient’s electronic healthcare record. Furthermore, in 82% of forensic psychiatric patients included in this study, all the information required to complete FoVOx could be extracted from the individual’s clinical record by a mental health professional unfamiliar with their case within 15 minutes. When one or more pieces of data were unknown, FoVOx generated a range of probability scores. For the lead clinician, FoVOx could be completed in around 1 minute for their most recent discharge and without recourse to clinical notes. Therefore, we conclude that FoVOx is feasible for clinicians familiar with a case; it is practical to use, requires no additional training, and minimal resource allocation. The brevity of the tool was repeatedly considered a strength in the qualitative clinician interviews.

We found a lack of any agreed practice around risk assessment at the point of discharge in this sample of UK forensic psychiatrists. Although these clinicians reported that they did complete a risk assessment, its nature varied. Some used existing tools completed within the prior 6 months, others a clinical assessment comprising a descriptive account in a psychiatric report, and some a multi-disciplinary discussion of risk.

The qualitative part of this investigation found that completing FoVOx was helpful to most clinicians. Benefits included improved information sharing with other agencies, reassurance for the clinical team, and identification of unaddressed or underweighted risk factors. FoVOx also assisted in guiding community management after discharge. In the future, 7 of the 11 clinicians reported that they would use FoVOx as part of their clinical practice. Benefits over existing tools used included its brevity, the ease with which information can be found, and how it clearly and transparently outlined a particular patient’s risk to other agencies who are less likely to be familiar with more detailed structured professional judgment tools currently used in forensic mental health settings. Clinicians who said that they would not use it in future provided mostly neutral feedback including that it did not provide any additional value. Specific criticisms were not unexpected, including the lack of dynamic factors, and the view that clinical risk assessment is more accurate than actuarial tools. Some of the themes identified when clinicians thought FoVOx was inaccurate may actually be indirectly measured by the tool. For example, close supervision was felt to reduce risk of recidivism. The FoVOx item regarding stay of over a year also lowers risk scores; patients with a longer inpatient stay are more likely to be in receipt of statutory supervision after discharge.

Overall, FoVOx scores were thought to be accurate in around two-thirds of the cases and, for some patients, clinicians revised their view of the risk of future violent re-offending to a higher level after being informed of the FoVOx scores, suggesting that probability-based FoVOx scores could assist clinical decision-making. Clinicians were more likely to underestimate risk compared to FoVOx scores. Reasons identified included FoVOx emphasizing risk factors underweighted by clinicians. Understandably, clinicians are most likely to focus on clinical factors which can be addressed, such as response to medical treatment, engagement in psychological therapy, and abstinence from substances abuse in the supported environment of hospital. Being informed of FoVOx scores thus may enable clinicians to rebalance the relative importance of these hospital-based clinical factors against static ones that are independently predictive of violent recidivism. Thus, using FoVOx routinely would prompt clinicians to keep these factors in mind, suggesting that FoVOx could be useful as an adjunct to clinical decision-making. However, local external validation is required to know the accuracy of FoVOx compared to clinician judgment. Without this, placing more weight on FoVOx scores would be unwarranted and clinical decision-making should take precedence. At the same time, awareness of the factors that might lead clinical teams to underestimate risk needs careful consideration—from weighting more recent factors, and structural (such as the need to maintain sustainable lengths of stay in secure services) and therapeutic factors.

One useful aspect of this feasibility work was to elicit views about the timing of FoVOx. As most factors are static and will not change during hospitalization, this provides some flexibility as to when it can be administered. At the point of discharge, patients are well-known to their clinical teams, and a more individualized and detailed risk formulation is likely to be available. FoVOx may be of more value if completed earlier in a patient’s pathway through the secure hospital system, possibly at the point of referral or gatekeeping into secure psychiatric care, or early in their admission. Completing the FoVOx tool at an earlier stage would allow for patient’s future risk to be stratified sooner, assist to guide their pathway through the secure hospital system and inform allocation of treatment resources.

One of the clinical implications of this study would be to integrate the FoVOx tool into patient care, for example by including it as part of their electronic patient healthcare record. Future work could compare FoVOx to other current risk assessment tools to compare acceptability and feasibility including the time taken to complete them, clinician satisfaction, and impact on patient care.

## Limitations

External validation was not conducted, which is a considerable challenge in forensic psychiatry due to patient numbers and event rates. If one assumes around 20% violent reoffending over 2 years, then around 500 forensic psychiatric discharges would be recommended for a validation study (18). This would likely require a large multi-centre study across different regions and/or nations. One limitation of the current investigation is that the outcome was based on clinical knowledge, and future work could triangulate this information with criminal records. When the available information was incomplete to complete FoVOx scoring, and a range of risk generated, we chose the higher value. An alternative would be to give an average value to missing variables to avoid potential over-estimation of risk.

A high proportion of cases (66%) reviewed were assigned to the medium risk category. If the majority of the patients are all assigned to the same risk class, this may reduce the clinical utility of the tool. Three of eleven clinicians felt that the low/medium/high categorization was unnecessary. In future research, solely using the probability score of reoffending can be examined.

Another limitation is that there may have been a positive bias to the qualitative data as the tool was developed by researchers locally, including one of the interview team. The study was also limited to forensic psychiatrists, and the views of other clinicians are necessary in further work.

Overall, the clinician views on FoVOx were consistently positive in many respects from informing decision-making to assisting risk communication. The novel features of FoVOx, including its brevity, online platform, and ease of use suggest that it can improve the risk assessment process in individuals detained in forensic psychiatric hospital.

## Data Availability Statement

Anonymized completed interview questionnaires and Service Evaluation proposal are available on request.

## Author Contributions

SF, RC, and AL designed the study. AL developed the standardized tool (**Appendix 1**). AL and OP completed FoVOx scoring of patients. RC, AL, and OP conducted clinician interviews. RC and OC completed thematic analysis. SF, RC, OP, SM, and AL wrote the paper.

## Funding

SF is funded by the Wellcome Trust.

## Conflict of Interest

SF and RC are authors on the original FoVOx paper. 

The authors declare that the research was conducted in the absence of any commercial or financial relationships that could be construed as a potential conflict of interest.
